# Synthesis and Characterization of Silica-Coated Oxyhydroxide Aluminum/Doped Polymer Nanocomposites: A Comparative Study and Its Application as a Sorbent

**DOI:** 10.3390/molecules25071520

**Published:** 2020-03-27

**Authors:** Inas A. Ahmed, H. S. Hussein, Ahmed H. Ragab, Najlaa S. Al-Radadi

**Affiliations:** 1 Department of Chemistry, Faculty of Science, King Khalid University, Abha 62224, Saudi Arabia; ahrejab@kku.edu.sa; 2 Chemical Engineering & Pilot Plant Department, Engineering Division, National Research Centre, Cairo 11865, Egypt; hala.hussein21@yahoo.com; 3 Department of Chemistry, Faculty of Science, Taibah University, Madinah Monawara 20012, Saudi Arabia; nsa@taibahu.edu.sa

**Keywords:** nano silica, nano-oxyhydroxide aluminum, polyaniline, nanocomposites, nickel, adsorption, equilibrium kinetics

## Abstract

The present investigation is a comparison study of two nanocomposites: Nano-silica-coated oxyhydroxide aluminum (SiO_2_–AlOOH; SCB) and nano-silica-coated oxyhydroxide aluminum doped with polyaniline (SiO_2_–AlOOH–PANI; SBDP). The prepared nanocomposites were evaluated by monitoring the elimination of heavy metal Ni(II) ions from aquatic solutions. The synthesized nanocomposites were analyzed and described by applying scanning electron microscopy (SEM), X-ray diffraction (XRD), energy dispersive X-ray spectroscopy (EDX), transmission electron microscopy (TEM), and Fourier transform infrared spectroscopy (FTIR) techniques, as well as Zeta potential distribution. In this study, two adsorbents were applied to investigate their adsorptive capacity to eliminate Ni(II) ions from aqueous solution. The obtained results revealed that SBDP nanocomposite has a higher negative zeta potential value (−47.2 mV) compared with SCB nanocomposite (−39.4 mV). The optimum adsorption was performed at pH 8, with approximately 94% adsorption for SCB and 97% adsorption for SBDP nanocomposites. The kinetics adsorption of Ni ions onto SCB and SBDP nanocomposites was studied by applying the pseudo first-order, pseudo second-order, and Mories–Weber models. The data revealed that the adsorption of Ni ions onto SCB and SBDP nanocomposites followed the pseudo second-order kinetic model. The equilibrium adsorption data were analyzed using three models: Langmuir, Freundlich, and Dubinin–Radusekevisch–Kanager Isotherm. It was concluded that the Langmuir isotherm fits the experimental results well for the SCB and SBDP nanocomposites. Thermodynamic data revealed that the adsorption process using SCB nanocomposites is an endothermic and spontaneous reaction. Meanwhile, the Ni ion sorption on SBDP nanocomposites is exothermic and spontaneous reaction.

## 1. Introduction

Water scarcity has become the most serious problem that is facing the world. Water is vital for the existence of all living beings and for the growth of countries. At the present time, the depletion of water is increasing quickly due to the acceleration of industrialization [1]. Hence, the environmental pollution problem is continuously threatening our world. These pollutants result in dangerous environmental and public health problems. Therefore, the elimination of heavy metals from wastewater is an essential matter. Polluting metal ions arise from water supplies, mine waters, and industrial effluent from processes such as electroplating and metal and wood processing [2]. Nickel contamination comes from industrial procedures, for instance, the manufacture of tableware, electroplates, connecters, lead frames, cadmium–nickel batteries, plastic, pigments, and fertilizers, as well as metallurgical and mining processes. Serious health problems, for example, skin dermatitis, damage to lungs and kidneys, and gastrointestinal distress, are caused by the ingestion of nickel through water. Moreover, nickel is a known carcinogen. Therefore, it is essential to create successful and economical methods to eliminate and/or recapture nickel. The Ni(II) concentration in wastewater has been investigated up to 130 mg/L. The nickel concentration can be close to 2–900 mg/L in plating rinse, and this be considered to be one of the most important toxic pollutants [3]. Several technologies have been suggested to eliminate heavy metal ions including adsorption [4], electrodialysis [5], chemical processes [6], filtration by membrane [7], biosorption [8], electrochemical methods [9], and ion-exchange resin [10]. Unfortunately, some of these methods are difficult to scale up or they are expensive to apply [11]. 

The adsorption process is the best, as it characterized by a highly effective regeneration ability and can be applied to various adsorbents. Adsorbents are similar to silica, hydroxyapatite, boehmite, chitosan, clay, polyaniline, polypyrrole, metal oxides, and activated carbon [12,13,14,15]. Nano alumina is one of the most amazing adsorbents for water treatment [16]. It was reported that nano aluminum oxyhydroxide has an excellent removal capacity [17]. Moreover, nano silica has been effectively utilized in water treatment. Nano silica’s efficiency as an adsorbent relies on (OH) groups in several situations. It acts as the center which the adsorbent interacts with the contaminant material [18]. Synthesis of polymers based on organic-inorganic materials is an auspicious method for the production of porous adsorbents with active functional groups. Lately, organic polymers like polyaniline and polypyrrole have been used as adsorbents for heavy metal discharge from contaminated water. These polymers are having lone pairs of electrons on nitrogen atoms that can coordinate with positive metal ions, resulting in spontaneous adsorption [19].

In the literature, the implementation of silica–polymer composites has been widely investigated [20]. Silica nanoparticles (NPs) are characterized by their chemical stability, diminished size, and ability to be modified chemically. Polymers such as polyaniline or polythiophene are considered to be conducting polymers that can be present under different oxidation states and react to outer stimulants by adjusting their physical properties like color, permeability, and conductivity [21]. Rathinam et al. [22] prepared a composite consisting of silica gel and polyaniline through in situ polymerization. It was investigated to evaluate the capability of the adsorbent to remove Cr(VI) ions from contaminated water. The data deduced that the maximum adsorption capacity of the polyaniline–SiO_2_ composite was 63.41 mg/g. Mohammad Nezhad et al. [23] presented the preparation of nano-boehmite/poly(methyl methacrylate nanocomposites with acceptable adsorption capacity for copper ions in contaminated water. The nano boehmite content was measured to be 1, 2, and 3 wt%, respectively, via an in situ polymerization process. Hence, the nanocomposites (boehmite content 3%, 120 min.) showed an excellent adsorption affinity for copper.

In this work, a comparative investigation is presented to evaluate the adsorption of Ni ions using two nanocomposites. The first is nano-silica-coated oxyhydroxide aluminum (SiO_2_–AlOOH; SCB). The second is nano-silica-coated oxyhydroxide aluminum doped with polyaniline (SiO_2_–AlOOH–PANI; SBDP). Both composites are examined by scanning electron microscopy (SEM), X-ray diffraction (XRD), energy dispersive X-ray spectroscopy (EDX), transmission electron microscopy (TEM), Fourier transform infrared spectroscopy (FTIR), and zeta potential. The nickel concentration is measured by a UV spectrophotometer. The kinetics of the adsorption of Ni ions onto SCB and SBDP nanocomposites are investigated using the pseudo first-order, pseudo second-order, and Mories–Weber kinetic models. Moreover, the equilibrium adsorption data are analyzed using three models: Langmuir, Freundlich, and Dubinin–Radusekevisch–Kanager Isotherm. Thermodynamic factors, enthalpy (ΔH^o^), entropy (ΔS^o^), and Gibbs free energy (ΔG^o^) are also evaluated.

## 2. Results and Discussion

### 2.1. Structural and Surface Characterization of the Adsorbent

#### 2.1.1. FT-IR Study

**Figure 1:**
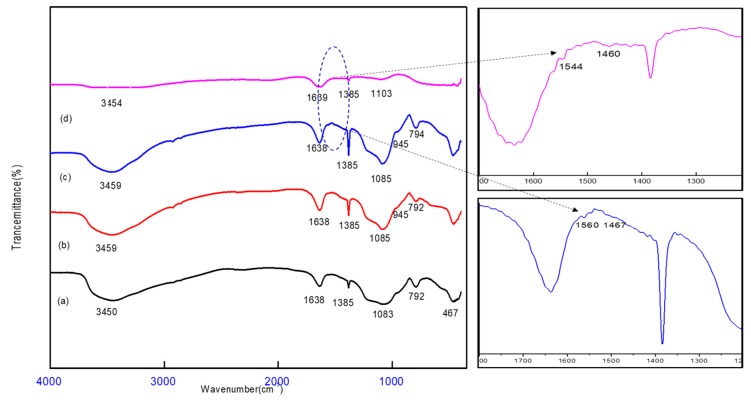
FTIR spectra of (**a**) nano-silica-coated oxyhydroxide aluminum (SCB), (**b**) Ni-sorbed SCB, (**c**) nano-silica-coated oxyhydroxide aluminum doped polyaniline (SBDP), (**d**) Ni-sorbed SBDP nanocomposite.

Figure 1 shows the Fourier transform-infrared spectra of the SCB and SBDP nanocomposites; Ni-sorbed SCB and Ni-sorbed SBDP are represented. All samples have similar peak positions at around 3450, 1638, 1358, 1085, 792, and 467 cm^−1^. The broad peak at 3450 cm^−1^ relates to the O–H stretching vibration, resulting from hydroxylation of OH via adsorbed water molecules [24]. The 1638 cm^−1^ peak relates to the bending vibration of H–O–H. The absorption peak at 1385 cm^−1^ concerns Al–OH and Si–OH [25]. Additionally, there is a strong peak at 1083 cm^−1^ which confirms the presence of Si–O–Si and Al–O–Al bonds [26,27]. The absorption band at 792cm^−1^ relates to the bending vibrations of the O–Al–O and Si–O–Si bending vibrations, respectively [28,29]. However, in Figure 1c, the SBDP nanocomposite shows other peaks, which are characteristic of the PANI layer—very small absorption bands around 1560 and 1467 cm^−1^ refer to CQN and CQC stretching vibrations from the quinoid and benzenoid structures of PANI, respectively [29]. The band corresponding to the C–N stretching mode at 1385 cm^−1^, which is related to benzenoid units overlaps with the stretching vibration peak of the Si–O–Si bond [30]. Moreover, the absorption band at 1103 cm^−1^ is specified to–NH vibration [31]. These characteristic peaks confirm that the coating of the PANI layer on the SCB and Ni-Sorbed SBDP nanocomposites was successful. Figure 1d shows a slight shifting of the bands from 3459,1544, 1467, 1085, and 792 cm^−1^ to 3454, 1560, 1460, 1103, and 794 cm^−1^, confirming the electrostatic interaction between Ni (II) ions and the SBDP nanocomposite. Meanwhile, Figure 1b reveals the shifting of the band from 3450 and 1083 cm^−1^ to 3459 and 1085 cm^−1^, proving the adsorption of Ni (II) ions on the SCB nanocomposite. 

#### 2.1.2. X-Ray Diffraction Study

**Figure 2:**
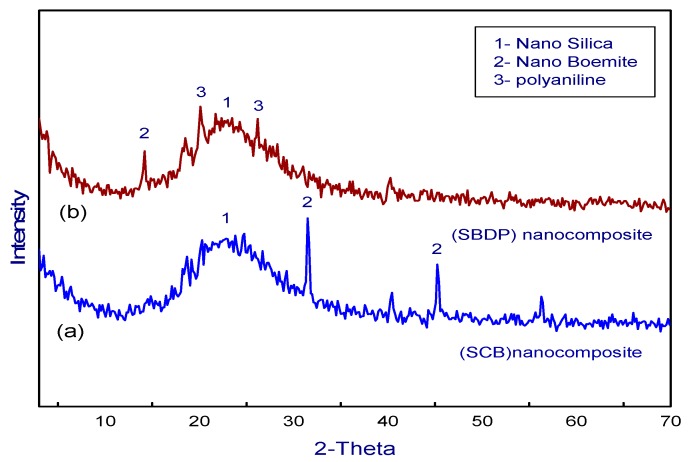
XRD pattern of (**a**) SCB, (**b**) SBDP nanocomposite

The formation of SCB and SBDP nanocomposites is also evident from the X-ray diffractograms (XRD) displayed in Figure 2. The data in Figure 2a indicate that the prepared SCB nanocomposite is amorphous in nature. The broad peak of 2θ = 22.4° explains that the obtained silica is an amorphous material [32]. The characteristic peaks at 32°and 45.5° represent AlOOH (oxyhydroxide aluminum) [33]. However, the peak beyond 22.4° in the diffractogram of nano silica is strong enough that the individual peaks of oxyhydroxide aluminum are not prominent in this region, indicating the coating of silica on oxyhydroxide aluminum. The X-ray diffractogram (Figure 2b) of the SBDP nanocomposite exhibits two specific peaks near 2θ = 20° and 25° respectively which indicate the periodicity parallel and perpendicular to the polymer chain [34]. The appearance of these peaks with the SBDP nanocomposite confirms the presence of polyaniline on the SCB nanocomposite. Moreover, the reduction in intensity of the oxyhydroxide aluminum peaks confirms the adherence of PANI to the SCB nanocomposite.

#### 2.1.3. SEM-EDX Study

**Figure 3:**
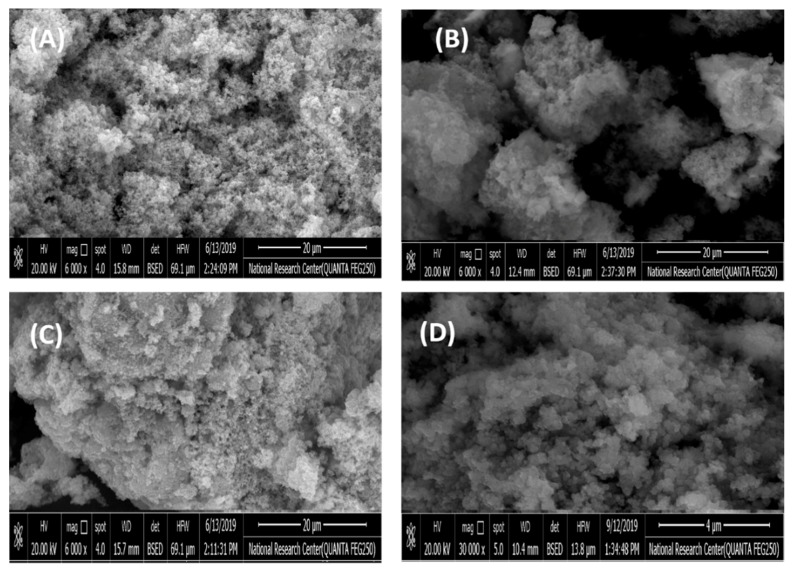
SEM of (**A**) SCB, (**B**) Ni-sorbed SCB, (**C**) SBDP, (**D**) Ni-sorbed SBDP nanocomposite.

The surface morphology of the SCB and SBDP nanocomposites was investigated via SEM-EDX analysis before and after binding with Ni(II). Figure 3 shows the surface morphology of the SCB, SBDP, Ni-sorbed SCB, and Ni-sorbed SBDP nanocomposites. As shown in Figure 3A,C, there is a rough porous texture with an adapted morphology for the adsorption of metal ions on the nanocomposite surface. The SEM images of the SCB nanocomposites show that the fluffy spherical particles of nano silica had a dense morphology, indicating that silica was successfully deposited on oxyhydroxide aluminum. However, as shown in Figure 3C, the SBDP nanocomposite has a rough, irregular surface related to polymerization that may have occurred in the internal channels, pores, and external surface of the SCB nanocomposite. Figure 3B,D, displaying the Ni-sorbed SCB and Ni-sorbed SBDP nanocomposites, shows that the grains of nanocomposites had become smooth and the surface had been covered by Ni ions after sorption.

**Figure 4:**
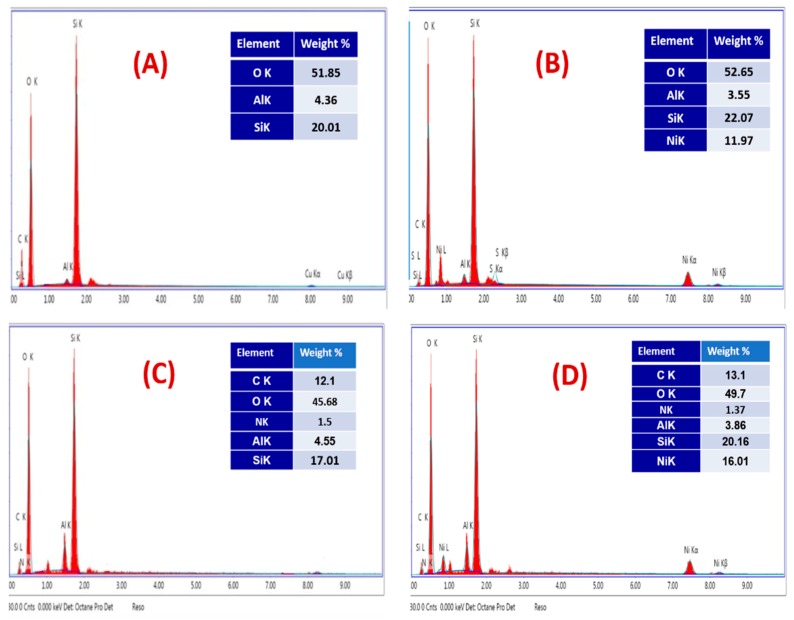
EDX of (**A**) SCB, (**B**) Ni-sorbed SCB, (**C**) SBDP, (**D**) Ni-sorbed SBDP nanocomposite.

The EDX analysis confirmed the adsorption of nickel ions onto the surfaces of both nanocomposites. The EDX spectra of SCB nanocomposite and the Ni-sorbed SCB nanocomposite are shown in Figure 4A,B. The EDX analysis of the SCB nanocomposite before adsorption shows the peaks corresponding to O, Si, and Al atoms (Figure 4A). After adsorption, strong nickel ion peaks appear in the EDX spectrum, which are related to Ni (II) metal ions. Meanwhile, the EDX spectra of SBDP and the Ni-sorbed SBDP nanocomposite are shown in Figure 4C,D. The EDX analysis of the SBDP nanocomposite before adsorption shows peaks concerning O, C, N, Si, and Al atoms (Figure 4C). After adsorption, a strong peak that is characteristic of Ni(II) appears (Figure 4D). Hence, EDX analysis ensures the trapping of Ni ions onto the surface of the SCB and SBDP nanocomposites via chelating and other linkages forming coordinate bonds. 

#### 2.1.4. Transition Electron Microscopy Study

**Figure 5:**
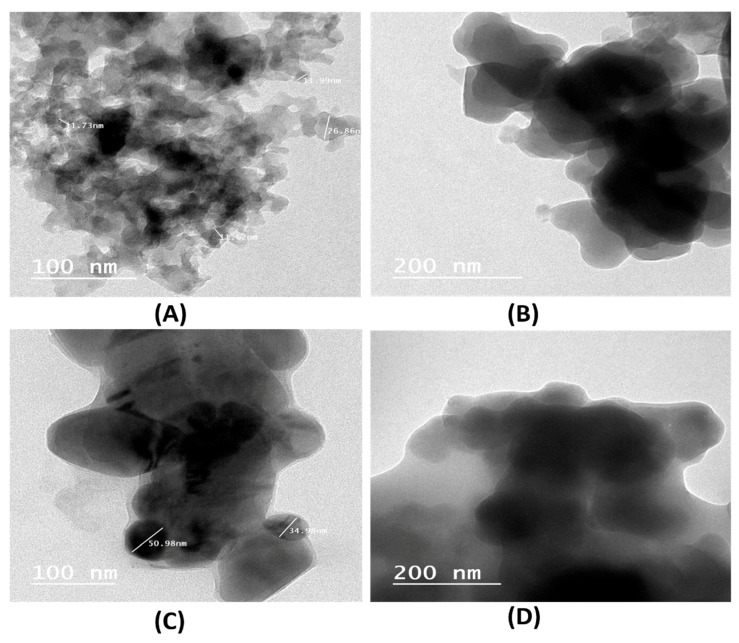
TEM analysis of the SCB nanocomposite (**A**) 100 nm view and (**B**) 200 nm view and the SBDP nanocomposite (**C**) 100 nm view and (**D**) 200 nm view.

The results of the TEM analysis of the SCB and SBDP nanocomposites are displayed in Figure 5. The TEM micrographs of the SCB nanocomposite in Figure 5B show a dense and porous structure with a crystal size of 11–26 nm. When densely doped with polyaniline (Figure 5C), the mean particle size of the SBDP nanocomposite increased to the range of a sheltered structure; this was related to the nano silica coating on nano oxyhydroxide aluminum.

However, after doping with polyaniline, as shown in Figure 5C, the mean particle size of the SBDP nanocomposite increased to the range of 34–50 nm. It is clear from Figure 5C that PANI largely covered the surface of the SCB nanocomposite, indicating a possible electrostatic attraction between PANI and the SCB nanocomposite.

### 2.2. Effect of pH

The pH of the solution is a very important factor that affects the removal efficiency of an adsorbent in wastewater treatment, since the efficiency of adsorption depends on the pH of the medium. pH change leads to alteration in the adsorbent properties and, consequently, the degree of ionization of heavy metals [35]. Moreover, the results of the zeta potential distribution of the SCB and SBDP nanocomposites are near to zero (−39.4 mV) and (−47.2 mV), respectively. These values elucidate that the particles have a negative zeta potential and may interact strongly with cationic additives. Also, it can be noticed that the SBDP nanocomposite has a higher negative zeta potential value compared with the SCB nanocomposite. 

**Figure 6:**
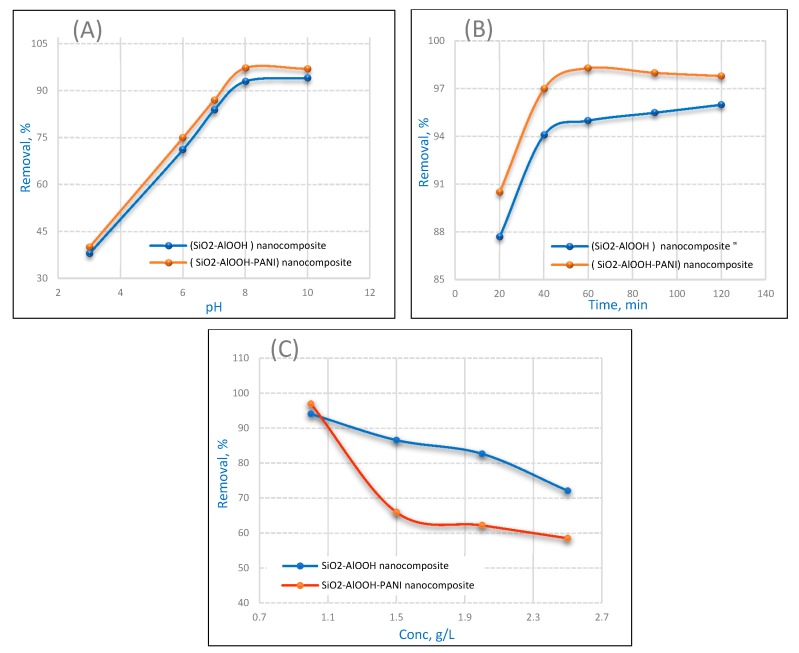
Influences of (**A**) pH, (**B**) contact time, and (**C**) initial Ni concentration, on the adsorption of Ni (II) by 0.3 g/50 mL nanocomposite at a pH of 8 and a contact time of 40 min.

The effect of the pH on the adsorption capacity of SCB and SBDP nanocomposites was studied over the pH range from 3 to 10. As depicted in Figure 6A, the data confirm a higher efficiency of adsorption of nickel(II) ions onto inorganic oxide adsorbent under an alkaline medium [36]. The adsorption of Ni(II) increased sharply up to pH 8 and did not show any significant change from 8 to 10. This might be explained on the basis of the fact that, in an acidic environment, the SCB and SBDP nanocomposites tend to promote positive charge on their conductive structures. Thus, under acidic medium, a considerable amount of electrostatic repulsion is created between the positively-charged.

SCB and SBDP nanocomposites and positive metal ions, which results in the adsorption efficiency of nanocomposites for Ni adsorption being minimized. As the pH increases, there is a reduction in competition between the positively-charged SCB and SBDP nanocomposites and the positive nickel ions, and as a result, adsorption behavior illustrates a gradual increase up to pH 8 [37,38]. Thus, for both SCB and SBDP nanocomposites, the optimum adsorption was performed at pH 8, with approximately 94% adsorption for SCB and 97% adsorption for SBDP nanocomposites, indicating that both SCB and SBDP nanocomposites are excellent adsorbent nanocomposites. It is clear that the adsorption abilities of SBDP nanocomposites are higher than those of SCB nanocomposites, which confirms that the adsorption effect is due to the polyaniline layer on the surface of the nanocomposites leading to the presence of a large number of functional groups. The sorption process in the basic medium may be explained with the following equations:

SCB^−^ + Ni (II)^+^ →SCB―Ni(II) (1)

SBDP^−^ + Ni (II)^+^ →SBDP―Ni(II). (2)

### 2.3. Effect of Contact Time

It is essential to know the effect of contact time to monitor the efficiency of an adsorbent. The removal percent of metal ions was computed at particular time intervals ranging from 20 to 120 min with an initial concentration of 1 g/L Ni (II) and pH 8 using 0.3 g/50 mL of adsorbent. Figure 6B illustrates the effect of the contact time on the percentage removal of Ni ions. It is obvious from the Figure 6B that the adsorption of Ni(II) increases steadily with an increase in time and reaches equilibrium at 60 min of contact time, with approximately 95% and 98% adsorption on the SCB and SBDP nanocomposites, respectively.

### 2.4. Effect of Ni(II) Ion Concentration

The influence of the Ni(II) concentration on adsorption was investigated from 1 to 2.5 g/L with 0.3 g/50 mL of nanocomposite, a contact time of 40 min, and a pH of 8, as represented in Figure 6C. It can be noticed that the removal of Ni(II) was enhanced with the decrease of the initial nickel ion concentration. Moreover, at a low concentration, the ratio of active surface sites on the composite surface to the total metal ions in the solution was high. Thus, all metal ions may interact with the adsorbent and potentially be ejected from the aqueous solution [39]. It is depicted that when the initial Ni (II) concentration increased, the absorption capacity of SBDP was lower compared with that of the SCB nanocomposite. Meanwhile, the SCB nanocomposite had a greater affinity for Ni ions. This is may be related to the lower particle size of the SBDP nanocomposite compared with the SCB nanocomposite, which tends to decrease the surface area. Moreover, there is some agglomeration with the polyaniline layer, as seen from the TEM analysis, which tends to decrease the absorption of Ni(II) ions.

The adsorption mechanism of Ni(II) ions on the SCB and SBDP nanocomposites can be elucidated as the reactivity of SCB nanocomposites is adjusted by its surface characteristics, for instance, composition and structural perfection. The adsorption properties of the SCB nanocomposite are based on the presence of the functional groups. In an aqueous environment, the SCB nanocomposites has a large number of surface hydroxyl groups. The hydroxyl ions (OH^−^) result in the negative charge of the nanocomposite; thus, the nanocomposite has the ability to capture the positive ions. The sorption process of nickel ions to hydrous solids or hydroxylated surfaces can be mechanistically explained as the surface complexation, which is mostly influenced by the electrostatic forces of attraction between nickel and the surface of the adsorbent. In addition, SCB nanocomposites have a high density of active sites due to their large surface area. Thus, the sorption of nickel to SCB nanocomposites is greatly increased. Meanwhile, SCB nanocomposites offer high efficiency for the removal of nickel ions. The adsorption abilities of SBDP nanocomposites are higher than those of SCB nanocomposites, and this is related to that the polyaniline layer on the surface of the nanocomposites, which has a large number of the functional groups that tend to increase the absorption of Ni(II) ions.

### 2.5. Kinetics Models

Adsorption kinetics explain the mechanism of the adsorption process, as well as the average uptake that is required for choosing the optimum parameters [40]. The optimum conditions were fixed as pH 8, SCB and SBDP nanocomposite mass of 0.3 g/50 mL, a contact time of 40 min, and an initial concentration of 1 g/L Ni(II). To investigate the mechanism of Ni^2+^ ion adsorption onto SCB and SBDP nanocomposites, three kinetic models were considered: Pseudo first-order reaction (PFORE), pseudo second-order reaction (PSORE), and Mories–Weber kinetic equation.

#### 2.5.1. Pseudo First-Order Reaction Kinetics

**Figure 7:**
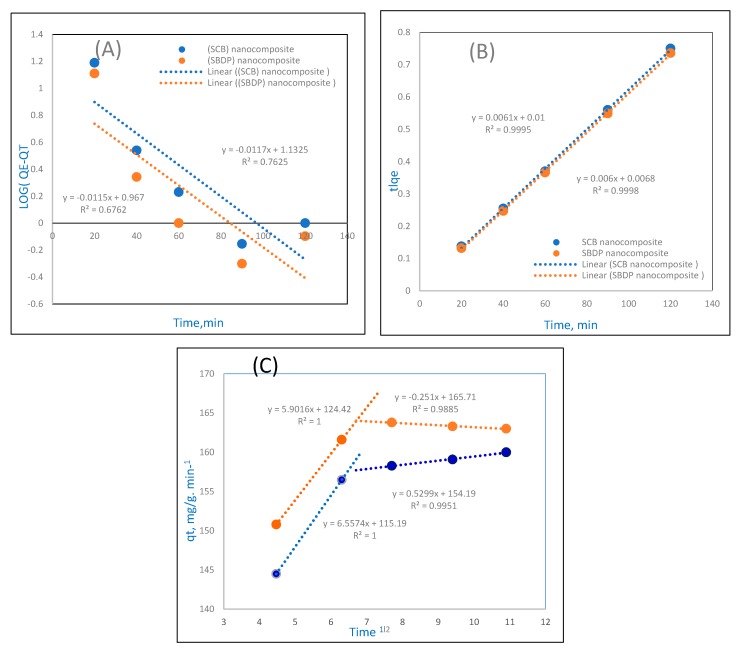
The adsorption kinetics: (**A**) pseudo first-order reaction (PFORE), (**B**) pseudo second-order reaction (PSORE), (**C**) Mories–Weber equation for Ni(II) adsorption on SCB and SBDP nanocomposites (sorption time 40 min; sorbent dosage 0.3 g/50 m L, pH = 8).

The equation for the PFORE reaction kinetics is shown below and represented in Figure 7 [41]. The PFORE equation is used for the rapid initial phase; the model equation is as follows:

Log (q_e_ − q_t_) − log q_e_ = −K_ads_ t/2.303 (3)

q_t_ mg/g is the adsorption capacity at time t, and k ads min^−1^ represents the rate constant of PFORE adsorption. In this study, from the Ni^2+^ ion adsorption onto SCB and SBDP nanocomposites, a linear relationship was obtained. The slope and intercept were calculated by plotting log (q_e_ − q_t_) versus t; the values of q_e_ and k _ads_ were calculated. Figure 7A is a graph of the PFORE kinetics. Table 1 shows that the PFORE correlation coefficients (R^2^) for the SCB and SBDP nanocomposites have low values. Moreover, the difference between the experimental and theoretical equilibrium adsorption (q_e_) is large, which means that the pseudo-first-order model has a poor fit.

#### 2.5.2. Pseudo Second-Order Reaction

The PSORE kinetic model [42] is shown in the following equation:

t/q = 1/K_2_q_e2_ + t/q_e_. (4)

k_2_ (g/mg/min) is the PSORE rate constant. As seen in Figure 7B, when t/qt is plotted versus t, the slopes and intercepts give the values of the rate constant (k_2_) and equilibrium adsorption capacity (q_e_). Moreover, the correlation coefficient (R^2^) values can be obtained. The results show high correlation coefficients (R^2^ = 0.9995 and 0.9998) for the SCB and SBDP nanocomposites. Also, these values are collected in Table 1. Regarding the calculated q_e_ values for both composites, they are also coincident with the obtained experimental data of the PSORE kinetics. These data mean that the adsorption for the SCB and SBDP nanocomposites are well suited to the pseudo second-order kinetics.

#### 2.5.3. Mories–Weber Kinetic Equation

The Mories–Weber Equation (7) explains the intraparticle mass transfer diffusion model [43] and is illustrated in Figure 7C:

q = K_d_ (t)^1/2^. (5)

q(g/g) represents the adsorbed metal ions, K_d_ is symbol of the intraparticle mass transfer diffusion rate constant, and t^1/2^ is the square root of time. Hence, if the adsorption data are coincident with the intraparticle diffusion, then it is the only shortened step. In Figure 7C, the Morris–Weber equation shows that the first part is linear, which may be related to the boundary layer effect, while the second part may be ascribed to the intraparticle diffusion effect [44]. This indicates that nearly all of the sorption takes place during the first 40 min with a definite linear direction, confirming that the porosity of the nanocomposites exceeds the resistance effects to intraparticle diffusion [45]. The intraparticle diffusion rate constant value K_d_ was estimated to be 0.5299 (g/g.min^−1^) for the SCB nanocomposite and 0.7598 (g/g.min^−1^) for the SBDP nanocomposite, which implies the movement of Ni ions towards the composite. From the data, it is obvious that the value of k_d_ for SBDP nanocomposites is higher compared with that of SCB nanocomposites. The kinetic modeling with the PFORE, PSORE, and Mories–Weber equations are represented Table 1.

### 2.6. Isotherm Model

Studying the adsorption isotherm will give useful information about the adsorption mechanisms, as well as the affinity of an adsorbent towards heavy metals ions and its surface properties [46]. The three isotherm models—the Langmuir, Freundlich, and Dubinin–Radusekevisch–Kanager—were applied to investigate the obtained data. The optimum conditions were adjusted to pH 8, a mass of 0.3 g/50 mL for the SCB and SBDP nanocomposites, a contact time of 40 min, and an initial concentration of 1 g/L Ni(II).

**Figure 8:**
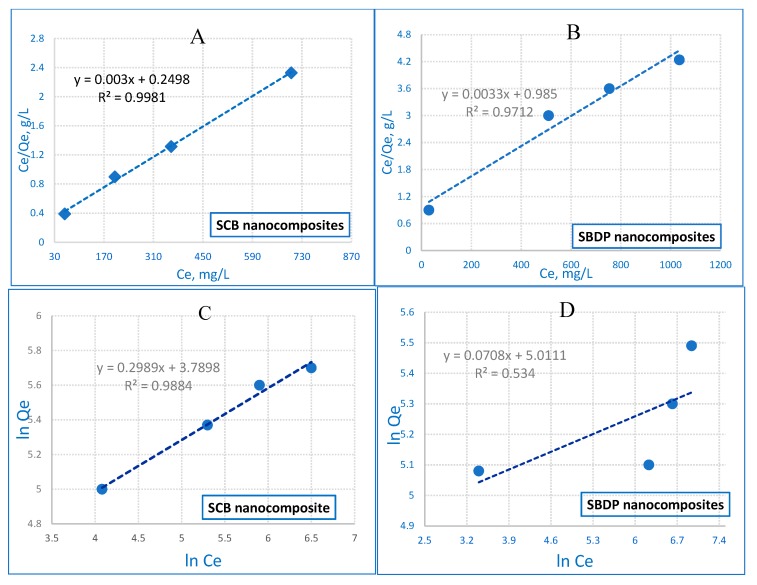
Langmuir adsorption for Ni ion removal on (**A**) SCB nanocomposites and (**B**) SBDP nanocomposites and Freundlich adsorption for Ni ion removal on (**C**) SCB nanocomposites and (**D**) SBDP nanocomposites (sorption time: 40 min; sorbent dosage: 0.3 g/50 mL, pH = 8).

The Langmuir isotherm is used to explain the adsorption of a substance on a homogenous surface with insignificant interaction between the adsorbed molecules [47]. The model postulates a uniform uptake on the surface with the highest adsorption, depending on the saturation level of the monolayer. In the following linear equation [48], the Langmuir model is represented:

Ce/q_e_ = 1/b.q_max_ + (1/q_max_).C_e_. (6)

b (L.mg^−1^) represents the monolayer adsorption capacity of the sorption heat, and q_max_(mg.g^−1^) is the maximum adsorption capacity. Figure 8A,B shows the Langmuir adsorption isotherm which is based on the monolayer adsorption of the adsorbate onto the adsorbent surface within the adsorption process. The Langmuir adsorption isotherm generally determines the equilibrium uptake of the homogeneous surface of adsorbents.

The Freundlich model is considered to be the earliest empirical equation that is compatible with the exponential distribution of active centers and is specific for heterogeneous surfaces [49,50]; the equation is as follows:

ln q_e_ = ln K_f_ + 1/n ln C_e_. (7)

K_f_ is the adsorption capacity, and n is the intensity. K_f_ is an important constant, it is known as a relative measure for the adsorption capacity. The value of n means a favorable adsorption extent. When the n value is higher than 1, this confirms the suitable nature of adsorption [51]. The results show that the Langmuir model is more suitable for fitting experimental data than the Freundlich model for both nanocomposites. The correlation coefficient (R^2^) values are shown in Table 2. The R^2^ values of the Langmuir plot for the SCB and SBDP nanocomposites are 0.9981 and 0.9712 higher than that of the Freundlich isotherm). Moreover, the adsorption capacities of 168.4 and 258.3 mg/g suggest that the removal of Ni ions expresses monolayer coverage on the SCB and SBDP nanocomposites’ surfaces. Consequently, the results fit the Langmuir model well.

#### Dubinin–Radusekevisch–Kanager Isotherm

Generally, this model is adequate for Gaussian energy distribution and adsorption processes on a heterogeneous surface. The equation of D-R as the following [52]:

ln q = ln q_(D-R)_ − ßε^2^ (8)

ε = RT ln(1 + 1/C_e_) (9)

q_(D-R)_(mg.g^−1^) is the theoretical adsorption capacity, ß is the activity coefficient (mol^2^ kJ^−2^) (the mean sorption energy), ε is the Polanyi potential, R is the ideal gas constant (0.008314 kJmol^−1^K^−1^), and T is the absolute temperature in K. E (kJ mol^−1^) is denoted as the free energy change:

E = 1/(2ß)^1/2^. (10)

The value of E can be applied to identify the kind of reaction. If E < 8 kJmol^−1^, the physical forces may influence the adsorption process. If E ranges from 8 to 16 kJmol^−1^, this means that the sorption occurs via chemical ion exchange. Moreover, the sorption process may be monitored by particle diffusion if E > 16 kJmol^−1^ [53]. The data obtained from the D-R model simulation are listed in Table 2. The E values are 0.711 and 0.714kJ mol^−1^ for Ni ion absorption onto the SCB and SBDP nanocomposites, respectively. Hence, if E < 8 kJmol^−1^, this means that sorption is processed by physical adsorption [54].

### 2.7. Sorption Thermodynamics

Thermodynamic parameters were applied to assess the spontaneity and heat change for the adsorption reaction. The results were collected at different temperatures (30, 40, and 50 °C). The thermodynamic parameters that comprise the standard enthalpy (∆H^o^), standard free energy (∆G^o^), and standard entropy (∆S^o^) were determined to define the thermodynamic action of the uptake of Ni ions adsorbed onto the SCB and SBDP nanocomposites. The thermodynamic parameters were estimated using the following equations [55,56]:

∆G^o^ = −RT ln K_d_ (11)

∆G^o^ = ∆H^o^ − T∆S^o^ (12)

LnK_d_ = −∆H^o^/RT + ∆S^o^/R (13)

where R is the gas constant (8.314 Jmol^−1^K^−1^), T is the absolute temperature (K), and K_d_ is the distribution coefficient.

The Gibbs free energy, thermodynamic parameter was computed by Equation (11). Also, ∆G^o^ can be determined from ∆H by applying Equation (12). The thermodynamic variables ∆S^o^ and ∆H^o^ were calculated using Equation (13) (from the intercept and slope). The data revealed that the amount of Ni ion uptake by both nanocomposites was minimized as the temperature was enhanced. Meanwhile, the increase in temperature will improve the solubility of contaminants in a bulk solution to a greater degree than the adsorbent particles [57]. The thermal parameters for the sorption of Ni ions on the SCB and SBDP nanocomposites are displayed in Table 3. The positive ∆H^o^ value means that the Ni sorption on the SCB nanocomposite is an endothermic process. The positive sign of the entropy change (∆S^o^) means that the adsorption of Ni ions onto the SCB nanocomposite is a random reaction. Moreover, the negative sign of ∆G^o^ means the adsorption of Ni ions onto SCB nanocomposite is feasible and spontaneous thermodynamically. 

The sorption appears to be chemi-sorption for ΔG^o^ values from −400 to −80 kJ mol^−1^, while it may be physical sorption when ΔG^o^ ranges from 20 to 0 kJ mol^−1^ [58]. Consequently, for the values of ΔG^o^ that are recorded in Table 3, the sorption of the SCB nanocomposite is physical sorption. This results in good agreement with the D-R isotherm. For the SBDP nanocomposite, the negative enthalpy value (∆H^o^) explains that the Ni ion sorption on the SBDP nanocomposite is exothermic. Moreover, the negative ΔG^o^ value suggests that the adsorption of Ni ions is spontaneous and feasible. As the temperature increases from 30 to 50 °C, the negative ΔG^o^ value decreases, elucidating that the adsorption process is spontaneous and more acceptable. The negative ΔS° shows that the molecules of Ni ions in the adsorbed phase of SBDP nanocomposite are more highly dispersed than contaminant molecules in bulk solution.

### 2.8. Cost Estimation per kg of the Adsorbent

The cost for the synthesis of the SBDP nanocomposite per kg is represented in Table 4. It was found to be nearly 13 USD per kg of adsorbent. The cost of one gram of commercial nanocarbon (Merck)and nano silica is nearly 456 and 198 USD, respectively, which means that SBDP is more economic and feasible for the application and substitution of high cost nano carbon and silica adsorbents [58].

Assume the cost of the raw material is 50% of the total production cost [59]. The total production cost is 2 × 6.3 = 12.7 USD/kg.

## 3. Experimental Procedures

### 3.1. Materials

The materials used included Al-dross produced by MCA company (Nag’ a Hammady, Egypt); hydrochloric acid (36%), sodium silicate, ammonium hydroxide (NH_4_OH), nickel sulfate (NiSO_4_ 6H_2_O), and aniline (ANI; extra pure 99%) from LOBA Chemic packed under nitrogen; ammonium persulfate (Aps; extra pure 98%) from Oxford lob chiem, India); and acetone ultrapure (99%) from Sigma (Cairo, Egypt). All chemicals were of commercial grade without purification. 

### 3.2. Synthesis of Nano Oxyhydroxide Aluminum

To synthesize nano oxyhydroxide aluminum, fine waste Al-dross (0.1 mm) was used. In this work, fine and pure oxyhydroxide aluminum was precipitated after treating by dilute commercial HCl. The synthesis conditions in terms of the concentration of the acid, leaching temperature, and time and solid/liquid ratio were optimized. The results indicate that the synthesis of Al-dross powder by a 1:4 concentration of HCl/tap water solution at 100 °C for 8 h yields a filtrate containing a maximum Al^3+^ ion content (~60%). The optimum conditions were adopted for the precipitation of pure gibbsite gel in the HCl-leaching filtrate by a 1:1 commercial ammonia solution at pH ~8 [60,61]. After that, the sample (gibbsite) was heated at 200 °C with a heating rate of 3 °C/min for a soaking time of 2 h to form nano oxyhydroxide aluminum [62]. Consequently, the synthesized nano oxyhydroxide aluminum was dried at a temperature of 105 °C. The transformation of Al-dross to oxyhydroxide aluminum is summarized in the following equation:

(14) $$Al\;dross\;\to \;Gibbsite\;\;Al{\left(OH\right)}_{3}\overset{200oC}{\to }\;oxyhydroxide\;aluminum\left(AlOOH\right)\;.$$

### 3.3. Synthesis of Nano-Silica-Coated Oxyhydroxide Aluminum Nanocomposite

**Figure 9:**
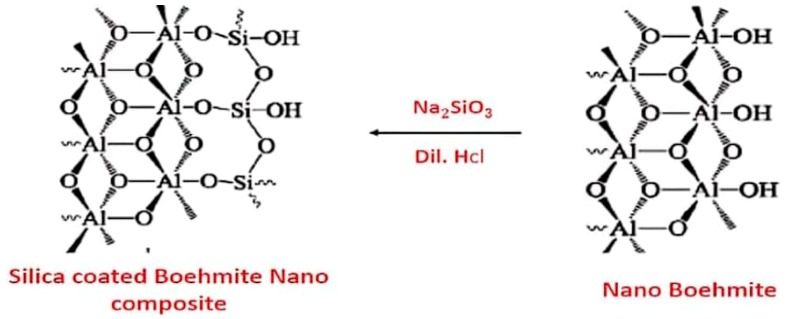
Nano-silica-coated oxyhydroxide aluminum (SCB) nanocomposites.

Nano-silica-coated oxyhydroxide aluminum (SCB) nanocomposites were prepared by adding 0.5 N of HCl acid to an aqueous solution containing oxyhydroxide aluminum and alkali metal silicate. The deposition of a sufficient amount of nano silica on oxyhydroxide aluminum was done to enhance the adsorption compatibility of oxyhydroxide aluminum to Ni metal ions. Oxyhydroxide aluminum powder (4 gm) was added to 50 mL of sodium silicate solution and then stirred for one hour to complete dispersion. Dilute HCl was added to the solution very quietly in order to ensure that the nano silica deposited on oxyhydroxide aluminum plates and did not form separately precipitated silica particles, with stirring for one hour at a temperature of 60 °C and pH 7. The nano silica was then precipitated on the surfaces of oxyhydroxide aluminum to form silica-coated oxyhydroxide aluminum nanocomposite [63]. The SCB nanocomposite was well rinsed and dried for 3 h at 50 °C. The nano-silica-coated oxyhydroxide aluminum (SCB) nanocomposite structure is shown in Figure 9.

### 3.4. Synthesis of Polyaniline

**Figure 10:**
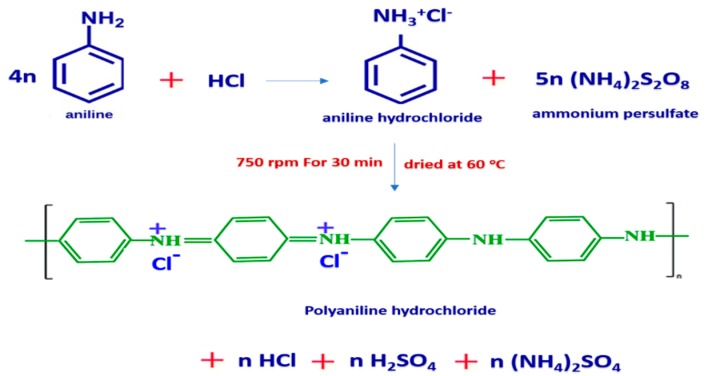
Polymerization of aniline hydrochloride to form polyaniline (emeraldine) hydrochloride.

About 1 mole of pure aniline (0.05 M) was dissolved in a known concentration of HCl (1 M). The required amount of ammonium persulfate (Aps) salt was dissolved in HCl (1 M) and added dropwise to the aniline solution. Then, the reaction was kept under stirring at 750 rpm for 30 min. Then, the precipitated dark green powder of PANI was filtered and rinsed by distilled water and acetone many times until the filtrate became colorless [64,65]. The washed precipitate was dried at 60 °C and ground to a fine powder in a mortar. Polymerization of aniline hydrochloride to form polyaniline (emeraldine) hydrochloride is shown in Figure 10.

### 3.5. Synthesis of Nano-Silica-Coated Oxyhydroxide Aluminum Doped Polyaniline Nanocomposite

**Figure 11:**
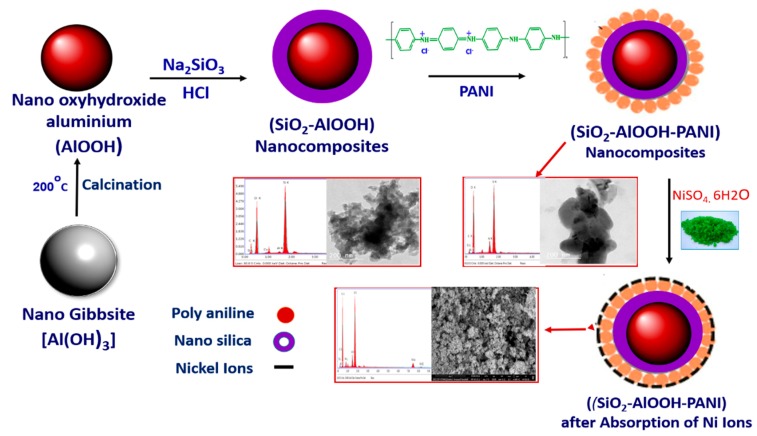
Flow chart of the synthesis process.

Nano-silica-coated oxyhydroxide aluminum doped polyaniline (SBDP) nanocomposite was prepared by suspending a mass (5 g) of the synthesized SCB nanocomposite powders into 200 mL of distilled water. The solution was stirred for 2 h at 303 K to form a homogeneous solution. Subsequently, 0.1 g of PANI in 100 mL of distilled water was added to this mixture slowly and stirred for 2 h to obtain a homogeneous solution of the doped composite. The resulting SBDP nanocomposite mixture was left for 48 h at 0–4 °C, filtered, washed several times with DD water, dried, and stored in an airtight container for further study. The obtained nanocomposites were subjected to characterization by scanning electron microscopy/energy dispersive X-ray spectroscopy (SEM/EDX, FEL, Eindhoven, Holland), X-ray diffraction (XRD, Philips, Amsterdam, Hollande), Fourier transform infrared spectroscopy (FTIR), and transmission electron microscopy (TEM). A flow chart of the synthesis process of silica-coated oxyhydroxide aluminum/doped polyaniline nanocomposite is shown in Figure 11.

### 3.6. Surface Characterization of the Nanocomposites

#### 3.6.1. Instruments

To characterize the obtained nanocomposites, FTIR-spectroscopy using potassium bromide (KBr) was conducted with a Genesis-II FT-IR spectrometer (ALT, San Diego, CA, USA) at a wavelength of 400–4000 cm^−1^. SEM was conducted using Inspect S (FEI Company, Eindhoven, Holland) equipped with an energy dispersive X-ray analyzer (EDX, Quanta 200, FEL, Eindhoven, Holland). The actual particle sizes of materials were measured by transmission electron microscopy (TEM) with the JEM-HR-2001 model (JEOL, Akishima, Japan) which was connected with an accelerating voltage of 200 kV. The mineralogical composition of the powdered materials was identified by X-ray diffraction (XRD) and recorded on a Philips PW 1050/70 diffractometer (Philips, Amsterdam, Hollande) using a Cu–Kα source with a post sample Kα filterant, a scanning speed of 1 s/step, a range of 5 to 50 (2θ°), and a resolution of 0.05°/step).

#### 3.6.2. Adsorption Studies of Nickel Ions

Nickel(II) sulfate hexahydrate (NiSO_4_ 6H_2_O)was used as the source of Ni (II) ions. The adsorption of nickel (II) were accomplished as follows: 50 mL of nickel(II) solution was stirred with nanocomposite under several conditions. The adsorption parameters, the contact time, pH, temperature, and initial concentration of Ni(II) adsorption behavior on both SCB and SBDP nanocomposites were investigated through batch experiments. The pH value of the nickel(II) was adjusted by using dil. H_2_SO_4_ and NaOH. The experimental work was carried out at room temperature (27 ± 1 °C). The variables of the adsorption studies were as follows: Nickel(II) concentrations: 1, 1.5, 2, and 2.5 g/L; initial pH of the solution: 3, 6, 7, 8, and 10; contact time: 20, 40, 60, 90, and 120 min; and adsorbent amount: 0.3 g/50 mL. The amount of metal adsorbed (q_e_) was determined by Equations (1)–(2) [66].

(15) $$\text{Adsorption Capacity}\;q_{e}=\frac{\left(C_{0}-C_{e}\right)V}{W}$$

(16) $$\mathrm{Adsorption}\;\%=\frac{\left(C_{0}-C_{e}\right)}{C_{0}}\times 100$$

where q_e_ (mg/g) denotes the equilibrium adsorption capacity, C_o_ and C_e_ are the initial and equilibrium concentrations (mg/L) of Ni ions, and V (L), and W (g) are the volume of the solution and weight of the adsorbent, respectively.

## 4. Conclusions

In the present study, SCB and SBDP nanocomposites for the elimination of Ni from aquatic solutions were successfully prepared and described through FTIR, SEM, XRD, TEM, and zeta potential distribution. The results of the present study clearly suggest that the SBDP nanocomposite has more negative charge on the surface than the SCB nanocomposite, and it can be effectively applied for Ni(II) elimination from wastewater. The sorption capacity is largely dependent on the heavy metal concentration and the pH of the solution. The adsorption of Ni(II) ions is maximized with as the pH increases. A pH of 8 was carefully chosen as the optimum pH for the adsorption of Ni(II) from aqueous solution. The kinetic process of Ni(II) adsorption onto SCB and SBDP nanocomposites fit the pseudo second-order rate equation well. Also, the equilibrium adsorption data for Ni(II) agreed with the Langmire adsorption isotherm model. The thermodynamic parameters were determined, and the reaction was shown to be endothermic and spontaneous for the adsorption of Ni(II) onto the SCB nanocomposite. Meanwhile, the Ni ion sorption on the SBDP nanocomposite was shown to be exothermic and spontaneous. The sorption of both SCB and SBDP nanocomposites is physical sorption. 

## Figures and Tables

**Table 1:** kinetic modeling with the PFORE, PSORE, and Mories–Weber equations.

Kinetic Models	Parameter	SCB	SBDP
PFORE	q_e_, exp(mg g^−1^)	160	163.8
	q_e_,cal(mg g^−1^)	13.5	9.2
	K_ads_(min^−1^)	0.0269	0.0264
	R^2^	0.9444	0.6762
PSORE	q_e_, cal(mg g^−1^)	163.13	165.3
	K_2_(g mg^−1^ min^−1^)	0.0037	0.0054
	R^2^	0.9995	0.9998
Mories–Weber	K_d_(mg g^−1^ min^0.5^)	0.5299	0.7598
	R^2^	0.9951	0.9885

**Table 2:** Sorption isotherms.

Kinetic *Isotherm*	Parameter	SCB	SBDP
Langmuir	q_e_, exp (mg g^−1^)	160	163.8
	q_e_,cal(mg g^−1^)	168.4	258.3
	K_L_(L mg^−1^)	0.0232	0.0040
	R^2^	0.9981	0.9712
Freundlich	K_F_(mol^n− 1^ L^n^ g^−1^)	101.5	156.8
	n	6.2	14.37
	R^2^	0.9884	0.5349
D-R model	E(kJ mol^−1^)	0.711	0.714
	q(D-R) (mg g^−1^)	788.3	244.6
	R^2^	0.9993	0.9992

**Table 3:** Thermal parameters for the adsorption of 1 g/L of Ni ions by 0.3g/50 mL of nanocomposite at a pH of 8 and a contact time of 40 min.

Parameter	T (K)	LnK_L_	∆H^o^ (KJ.mol^−1^)	∆S^o^ (J.mol^−1^.K^−1^)	∆G^o^ (kJ.mol^−1^)	R^2^
Ni^+2^/SCB	303	7.11	8.16	85.55	−17.91	0.9998
	313	7.3			−18.99	
	323	7.4			−19.87	
Ni^+2^/SBDP	300	8.58	−83.19	−202.77	−21.62	0.9968
	313	6.77			−17. 59	
	323	5.53			−14.85	

**Table 4:** Cost estimation per kg of the adsorbent (SBDP nanocomposite).

Raw Material	Amount	Total Price (USD)
Al-dross powder	500 g	No cost (waste)
Commercial HCl	3.00 L	6.20
Sodium silicate	1.00 L	0.01
Aniline	20 g	0.06
Sodium per sulphate	10 g	0.10
Net cost		6.3 (USD)
